# Risk factors for hematemesis in Hoima and Buliisa Districts, Western Uganda, September-October 2015

**DOI:** 10.11604/pamj.2017.28.215.12395

**Published:** 2017-11-08

**Authors:** Steven Ndugwa Kabwama, Richardson Mafigiri, Stephen Balinandi, Atek Kagirita, Alex Ario Riolexus, Bao-Ping Zhu

**Affiliations:** 1 Uganda Public Health Fellowship Program, Field Epidemiology Track, Ministry of Health, Kampala, Uganda; 2 US Centers for Disease Control and Prevention, Kampala, Uganda; 3 Central Public Health Laboratories, Ministry of Health, Kampala, Uganda

**Keywords:** Hematemesis, outbreak, case-control, Uganda

## Abstract

**Introduction:**

On 17 September 2015, Buliisa District Health Office reported multiple deaths due to haemorrhage to the Uganda Ministry of Health. We conducted an investigation to verify the existence of an outbreak and to identify the disease nature, mode of transmission and risk factors.

**Methods:**

We defined a suspected case as onset of hematemesis between 1 June 2015 and 15 October 2015 in a resident of Hoima, Buliisa or neighbouring districts. We identified cases by reviewing medical records and actively searching in the community. We interviewed case-patients and health-care workers and performed descriptive epidemiology to generate hypotheses on possible exposures. In a case-control study we compared exposures between 21 cases and 81 controls, matched by age (± 10 years), sex and village of residence. We collected 22 biological specimens from 19 case-patients to test for Viral Haemorrhagic Fevers (VHF). We analysed the data using the Mantel-Haenszel method to account for the matched study design.

**Results:**

We identified 56 cases with onset from June to October (attack rate 15/100,000 in Buliisa District and 5.2/100,000 in Hoima District). The age-specific attack rate was highest in persons aged 31-60 years (15/100,000 in Hoima and 47/100,000 in Buliisa); no persons below 15 years of age had the illness. In the case-control study, 42% (5/12) of cases vs. 0.0% (0/77) of controls had liver disease (OR_M-H_ = ∞; 95%CI = 3.7-∞); 71% (10/14) of cases vs. 35% (28/81) of controls had ulcer disease (OR_M-H_ = 13; 95% CI = 1.6-98); 27% (3/11) of cases vs. 14% (11/81) of controls used indomethacin prior to disease onset (OR_M-H_ = 6.0; 95% CI = 1.0-36). None of the blood samples were positive for any of the VHFs.

**Conclusion:**

This reported cluster of hematemesis illness was due to predisposing conditions and use of Non-Steroidal Anti-inflammatory Drugs (NSAID). Health education should be conducted on the danger of NSAIDs misuse, especially in persons with pre-disposing conditions.

## Introduction

Hematemesis refers to the vomiting of blood, which indicates acute gastro-intestinal bleeding. Common causes of upper gastro-intestinal bleeding include peptic ulcers, cirrhosis with oesophageal or gastric varices, gastritis and esophagitis of various etiology, Mallory-Weis tears and malignancy [1]. Hematemesis could also be a symptom of chronic infection with Schistosoma Mansoni [2]. A loss of gastro-intestinal micro-vascular integrity is also a characteristic of Viral Haemorrhagic Fevers (VHF) such as Ebola, Marburg, Lassa fever, Yellow fever and Rift valley fever [3]; therefore hematemesis might indicate a VHF especially if it is accompanied by fever and bleeding from other orifices. On 17 September 2015 the Ministry of Health of Uganda received a report from Buliisa District Health Office in western Uganda about a cluster of a mysterious fatal disease in Butiaba Sub-county. The dominant symptom was hematemesis while some patients had fever. By 24 September 2015, there had been 4 deaths. Since outbreaks of VHFs have occurred in nearby districts in the past [4-6], VHF was one of the differential diagnoses for this disease outbreak initially. We conducted an investigation to establish the existence of an outbreak, verify the diagnosis of the disease and inform public health interventions.

## Methods

Hoima and Buliisa Districts are located in the Western region of Uganda on the shores of Lake Albert. According to the 2014 national census, Hoima has a population of 573,903 while Buliisa District has a population of 113,569 [7]. We conducted the investigation between 3 and 17 October 2015. We defined a suspected case as onset of hematemesis in a resident of Hoima, Buliisa or another neighbouring district from 1 June 2015 onward. We found cases by reviewing health facility records from June 2015 to September 2015. We also visited affected communities to search for cases using the case definition. We also interviewed village health team members, healthcare workers and case patients who sought care at the health facilities. We analyzed the case-patients' line-list data to elucidate the distribution of case-persons by person, place and time. Based on the results of the descriptive epidemiology, we formulated hypotheses about potential exposures. In the assessment of alcohol use as a potential exposure, we asked patients about the nature of alcohol they usually consumed and the average amount in one sitting. The alcohol consumed was quantified as number of standard drinks whereby one standard drink was equivalent to 285ml of beer or 30ml of distilled [8]. People who took more than 6 standard drinks were categorised as exposed. We conducted a case-control study to test alcohol intake as a possible risk factor for hematemesis among other hypotheses. In the case-control study, a case was defined as onset of hematemesis in a person during July to October 2015. A control-person was an individual without any history of hematemesis. Control-persons were matched to cases according to sex, age (± 10 years) and village of residence, with a case-to-control ratio of 1:4.

**Laboratory investigation**: For 2 of the case-patients, an ultrasound device was used to visualize the size and structure of the internal organs around the abdomen. We collected stool samples from 3 case-patients and conducted microscopic identification of Schistosoma eggs in the sample. Blood was drawn from 1 of these 2 case-patients to perform liver function tests and a complete blood count. Multiple biological specimens (17 whole blood, 3 biopsies and 2 rectal swabs) from 19 patients were collected and tested for a battery of VHFs (including Ebola, Marburg, Crimean-Congo haemorrhagic fever (CCHF) and Rift Valley fever (RVF) viruses) using Polymerase Chain Reaction (PCR), histopathology and bacterial examinations. PCR tests were conducted at the Centers for Disease Control and Prevention/Uganda Virus Research Institute (UVRI) VHF laboratory, Entebbe, Uganda, while the bacteriological tests were performed at the Medical Research Council Microbiology Laboratory, also based at UVRI. These laboratories are national reference centres for viral and bacteriological diagnostics. The VHF diagnostic protocols used have been described before and were routinely used to confirm previous VHF outbreaks in Uganda [9]. Histopathology testing was done at the Centers for Disease Control and Prevention, Atlanta, USA.

**Statistical analysis**: Using population data from the national census [7] and the data provided by the Uganda Bureau of Statistics 2015 [10] on the age and sex distributions of the population, we calculated the attack rate by age and sex. In analysing the data from the case-control study, we used the Mantel-Haenszel method to estimate odds ratios (OR) and their confidence intervals (CI) to account for the matched study design.

**Ethical considerations**: The Ministry of Health of Uganda gave the directive and approval to investigate this outbreak. The Office of the Associate Director for Science, CDC/Uganda, determined that this activity was not human subjects research and its primary intent was public health practice or a disease control activity (specifically, epidemic or endemic disease control activity). Verbal informed consent was obtained from the participants before the start of each interview. Study participants were told that their participation was entirely voluntary and their refusal to answer any or all of the questions would not result in any negative consequences. Participants identified as patients were referred for free treatment at Hoima Regional Referral Hospital. To protect participants' confidentiality, personal information were de-identified during data analysis and the interview forms were locked up.

## Results

**Figure 1:**
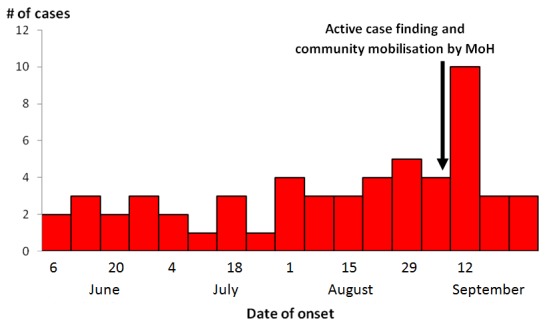
Epidemic curve showing number of hematemesis cases in Hoima and Buliisa Districts by week of onset, June-October 2015

**Figure 2:**
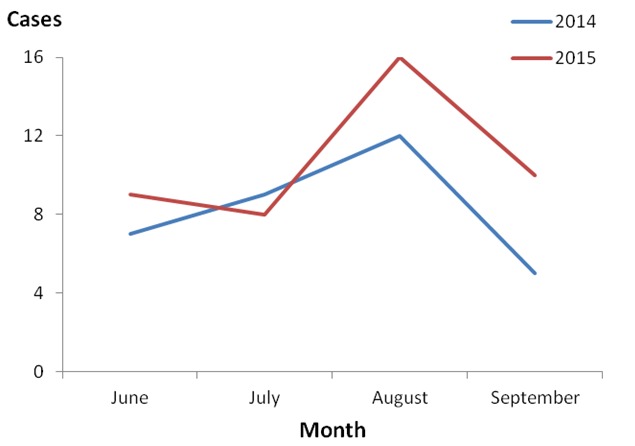
Number of hematemesis cases, by month, June-September: Hoima and Buliisa Districts, 2014 and 2015

Our active case-finding identified 56 hematemesis cases with disease onset between June and October 2015. The attack rate in Buliisa District was 15/100,000 while that in Hoima District was 5.2/100,000. The epidemic curve shows that approximately 3 cases occurred every week from June 2015 to September 2015 (Figure 1). The peak occurred in the middle of September after the Ministry of Health started community mobilization and active case-finding. The data from the review of records in both Hoima and Buliisa Districts showed an average of about 8 cases recorded for every month (Figure 2). For both years 2014 and 2015, the month of August had the highest number of cases. More cases occurred in August and September 2015, compared with the same period in 2014. Overall, we observed no consistent patterns in the trends of the case counts in the affected districts. The attack rate was higher in Hoima compared to Buliisa District. In both districts, persons aged 31-60 years had the highest attack rate followed by adults 18-30 years (Table 1). The attack rates in all age groups were higher in Buliisa compared to Hoima except the > 60 age group where Buliisa did not register any cases. no cases occurred among persons < 15 years of age in either district. Otherwise, no remarkable patterns were observed regarding the age-group distributions of the cases. In Hoima District, women had a higher attack rate than men while in Buliisa District men had a higher attack rate.

**Table 1:** Attack rates of hematemesis by age-group and sex during an outbreak in Hoima and Buliisa districts, Western Uganda, June–October 2015

	N		Population		Attack rate (100,000)	
	Hoima	Buliisa	Hoima	Buliisa	Hoima	Buliisa
**Age (years)**						
< 18	1	1	321,960	63,712	0.31	1.6
18-30	12	6	127,980	25,326	9.4	24
31-60	15	9	97,563	19,307	15	47
> 60	2	0	26,400	5,224	7.6	0
**Sex**						
Female	19	4	287,198	53,361	6.6	7.5
Male	11	12	286,705	51,573	3.8	23

**Case-control study**: In the case-control study, we found statistically significant associations between self-reported history of liver disease and hematemesis and self-reported history of ulcer disease and hematemesis (Table 2). Among the medications taken before case-patients' onset of illness, use of indomethacin was significantly associated with the illness.

**Table 2:** Association between risk factors and onset of hematemesis during an outbreak in Hoima and Buliisa Districts, Western Uganda, June-October 2015

Pre-existing condition and substance use	Number		% Exposed		OR_M-H_ (95% CI)
	CasesN=20	ControlsN=81	Cases	Controls	
**Pre-existing condition**					
Liver disease	5/12	0/77	42	0	∞ (3.7-∞^+^)
Ulcers	10/14	28/81	71	35	13 (1.6-98)
History of tuberculosis	3/13	2/80	23	3	5.0 (0.83-31)
Schistomiasis	7/12	25/79	58	32	2.7 (0.55-14)
No conditions reported	0/19	34/76	0	45	0 (Undefined^++^)
**Substance used before case-patient’s onset**					
Indomethacin	3/11	11/81	27	14	6.0 (1.0-36)
Ibuprofen	2/11	7/81	18	8.6	2.0 (0.35-12)
Alcohol use	2/3	20/37	67	55	1.8 (0.15-22)

+ Fisher’s exact confidence interval.

++ Confidence interval was undefined because of the zero-cell

**Clinical and laboratory investigation**: Of 2 case-patients that underwent ultrasonography, 1 showed advanced micro-nodular liver cirrhosis with tense ascites and gross splenomegaly. The differential diagnoses included severe acute large bowel inflammatory disease (amoebiasis, ulcerative colitis, crohn's disease, or schistosomiasis). This patient had low total protein 61 g/L (range 63-83) and elevated serum aspartate transferase 48 U/L (range 0-40). The complete blood count revealed a normal white blood cell count 4.9 103/uL (range 3.0-15), normal red blood cell count 3.0 106/uL (range 2.5-5.5), normal platelet count 152 103/uL (range 50-400) and low haemoglobin 7.8 g/dL (range 8.0-17). The abdominal scan of the second case-patient showed thickened gastric walls (up to 0.68cm) and an excessive amount of gastric gas. The liver, spleen, gall bladder and kidneys appeared normal and no abdominal masses or peritoneal effusion were seen. The findings were suggestive of gastroenteritis and ruled out complicated ulcers. Of the 3 stool samples examined by microscopy, one had Schistosoma ova. All samples tested were negative for Ebola, Marburg, CCHF and RVF by molecular testing. Testing of the rectal swabs using general culture for microbiological pathogens did not show any significant bacterial growth. Immunohistochemical testing on the submitted biopsies was also negative for Ebola and Marburg viruses, malaria and typhus group rickettsia.

## Discussion

Our investigation of the reported hematemesis outbreak ruled out a true outbreak. The surge in case count was likely to have been due to increased awareness and active case finding in the community after the initial cluster of cases were reported. Specifically, laboratory investigations did not point to VHF as the cause of this outbreak, as was initially suspected. Our data suggested that this illness cluster might have been caused by a combination of predisposing conditions (including liver disease, schistosomiasis and ulcer) and the misuse of NSAIDs (such as indomethacin and ibuprofen) that are known to trigger bleeding in patients with a bleeding tendency. In reviewing detailed clinical information of 31 patients with a post-mortem diagnosis of peptic ulcers, Felix and Stahlgren found that hematemesis was the initial symptom for 35% (11/31) of the patients; autopsy showed that 58% (18/31) of the patients had an ulcer located in the duodenum and 12 patients had an ulcer in the stomach and 10 of those patients had a bleeding ulcer [11]. In another report of 52 cases that had bleeding gastric and duodenal ulcers over 9½ years, 6 of the cases resulted in deaths; all 6 deceased persons were between the ages of 35 and 60 years and 4 had a history of hematemesis [12]. Similarly, the use of anti-inflammatory drugs is known to increase the risk for upper gastro-intestinal bleeding [13]. Pathophysiologically, NSAIDs block the synthesis of prostaglandins, which promote the production of mucus that protects the lining of the gut from ulceration [14], leading to hematemesis. Epidemiologically, indomethacin use has been associated with bleeding and other adverse effects of the gastro-intestinal tract. In a Swedish study, among 18 patients treated using Indomethacin for 6 months, 8 (44%) developed gastrointestinal related complications and 1 died [15]. Also, a case-control study conducted in the United Kingdom which consisted of 1457 cases of upper gastro-intestinal bleeding and 10000 control subjects, the relative risk associated with the use of NSAIDs was 4.7 (95% CI: 3.8-5.7) [13].

The clinical presentation of a distended abdomen among some of the case patients is of particular interest. Although in this investigation we did not find a statistically significant association between the history of self-reported schistosomiasis and hematemesis, the presentation of some cases with distended abdomens and the fact that one of three patients' stool specimens had positive identification of Schistosoma ova indicate that some of the case-patients have schistosomiasis. The shores of lake Albert where both Buliisa and Hoima Districts are located have the highest prevalence of symptomatic and asymptomatic Mansoni S. in Uganda [16]. The enlarged abdomen could be a result of splenomegaly from chronic infection with Mansoni S. One study revealed that even a low community prevalence of infection with Mansoni S. can influence hepatosplenic morbidity [17]. In a case series in Brazil, hematemesis frequently occurred in patients with schistosomal splenomegaly [18]. Drug administration of praziquantel either singly [19] or in combination [20] should be implemented to treat any existing schistosomal infections. Also, because infection with the parasites is a result of contact with contaminated water, the sanitation should be improved in this community. The improvement of sanitation have been shown to reduce schistosomiasis related morbidity by as much as 77% [21]. This investigation was initiated because the symptoms of initial patients were suggestive of VHFs [3]. Although VHFs were eventually ruled out, the investigation and response served as a “live fire exercise” for the surveillance and response capacity of the Ministry of Health. In this age when the global public health system is constantly challenged by emerging and re-emerging infectious diseases as well as new cycles of pandemics and threats of bioterrorism [22], such a “live fire exercise” helps to continuously improve the emergency response system. Without a timely and effective response, public health emergencies of international concern could quickly spiral out of control, as were exemplified by the Ebola epidemic in West Africa [23] and SARS pandemic in China [24]. Conversely, rapid response and control in Uganda and Nigeria were shown to effectively contain these emergencies and prevent them from becoming a public health crisis [25, 26].

This investigation also revealed widespread alcohol use in this population, which is consistent with findings from previous studies showing high prevalence of alcohol use among people that reside near water bodies in Uganda [27-29]. Alcohol intake of more than 6 standard drinks has been shown to be a significant predictor of future liver disease [30]. The high levels of alcohol use could explain the high prevalence of liver disease in this population, which is a major risk factor for hematemesis [31]. Epidemiological investigations have also revealed that among persons who use NSAIDS, the risk of acute upper gastro intestinal bleeding increases with the level of alcohol consumed [32]. Pharmacies in the area should warn their clients on the risk of gastrointestinal bleeding when NSAIDS are taken with alcohol. In Uganda, many prescription and non-prescription medicines are sold by both licensed and unlicensed drug shops. Although there is legislation that specifies which medicines could be sold as non-prescription drugs, a wide gap still exists between policy and implementation [33]. This combination of a society with high levels of alcohol use and wide availability of over-the-counter medicines increases the risk of complications that arise from use of drugs such as NSAIDs, especially among persons with pre-existing conditions such as ulcers, schistosomiasis, liver disease and tuberculosis. People in rural communities in Uganda have been shown to have low access to health care [34], low satisfaction with and poor perceived accessibility of the health care services [35]. Because of poor access to healthcare, it is possible that persons in our investigation were forced to self-medicate on drugs such as NSAIDs, which elevated their risk of developing hematemesis. Targeted interventions that address the widespread alcohol use, increase access to health care and health education on the dangers of NSAID use should be carried out for persons in this community and fisher folk in general.

## Conclusion

The increase in the number of cases of hematemesis in September 2015 was likely to have been due to enhanced surveillance. The hematemesis illness appeared to be endemic in this community and is likely to have been due to predisposing conditions (such as liver disease, schistosomiasis and ulcer), combined with the use of NSAID. We recommend that health education be conducted on the danger of misuse of NSAIDs, especially in persons with pre-disposing conditions. **Limitations**: in our investigation, the diagnoses of ulcer, tuberculosis, liver disease and other conditions were based on self-reports, which could cause bias. Also, for patients who had died before our investigation, we relied on proxy interviews of their family members and friends, potentially resulting in information bias.

### What is known about this topic

- Hematemesis indicates acute gastro-intestinal bleeding which could be due to peptic ulcers, cirrhosis with oesophageal or gastric varices, gastritis and esophagitis of various etiology, Mallory-Weis tears and malignancy;
- Conditions that present with hematemesis may indicate outbreaks of viral hemorrhagic fevers;
- People that reside around the shores of Lake Albert have been shown to have high prevalence of schistosomiasis infection in Uganda.

### What this study adds

- Pre-existing conditions such as ulcer diseases combined with use of Non-steroidal anti-inflammatory drugs can aggravate hematemesis;
- Alcohol use and schistosomiasis may also increase the risk of hematemesis;
- Swift and prompt investigation into conditions that present with hematemesis can be an effective way of preventing the spiralling of public health emergencies of international concern as was evidenced in the Ebola outbreak in West Africa.

## Competing interests

The authors declare no competing interests.
